# The Comparison of the Effect of Adding Empagliflozin to the Medication Regimen on Peripheral Neuropathy in Patients With Type II Diabetes: A Double Blind Randomised Clinical Trial

**DOI:** 10.1002/edm2.70128

**Published:** 2025-11-20

**Authors:** Mohammadreza Vafaeinasab, Fatemeh Mirzaei Malekabad, Amidodin Khatibi, Akram Ghadiri‐Anari, Marzieh Zare Bidaki, Raihane Azizi

**Affiliations:** ^1^ Department of Physical Medicine and Rehabilitation Shahid Sadoughi University of Medical Sciences Yazd Iran; ^2^ Department of Internal Medicine Shahid Sadoughi University of Medical Sciences Yazd Iran; ^3^ Diabetes Research Center Shahid Sadoughi University of Medical Sciences Yazd Iran

**Keywords:** diabetes mellitus, empagliflozin, peripheral neuropathy, SGLT2 inhibitors

## Abstract

**Introduction:**

Diabetes is the common metabolic disorder. Empagliflozin is a new medication that acts as a sodium‐glucose cotransporter 2 (SGLT2) inhibitor. It promotes increased glucose excretion through urine, which helps improve blood sugar control, enhances glucose metabolism, and reduces glucotoxicity and insulin resistance. This study aimed to investigate and compare the effects of adding empagliflozin to a medication regimen on peripheral neuropathy in diabetes.

**Materials and Methods:**

This study involved 50 patients with diabetic neuropathy, confirmed by the Michigan Neuropathy Screening Instrument, who were randomly assigned to two groups. The control group continued their previous medication regimen with a placebo, while the treatment group added 10 mg of empagliflozin daily to their existing regimen. At the start of the study, fasting blood glucose, haemoglobin A1c, serum creatinine levels and electrophysiological findings (including amplitude, latency and nerve conduction velocity) were assessed for all patients. These parameters were re‐evaluated at the end of the 20‐week intervention, and the results were analysed using statistical software.

**Results:**

At the end of the study, there was a significant reduction in both haemoglobin A1c and fasting blood glucose levels in the treatment group. Serum creatinine levels also significantly decreased. eGFR (estimated glomerular filtration rate) increased in the treatment group. In the Michigan Neuropathy Screening Instrument, the oral test score improved in the treatment group, but no significant difference was observed in the clinical examination scores. Additionally, electrophysiological testing showed a significant improvement in nerve conduction velocity in the treatment group. Latency also decreased in the treatment group, but no significant difference was observed in amplitude between the two groups.

**Conclusion:**

Empagliflozin reduces blood glucose levels and improves kidney filtration function. It also enhances clinical symptoms and nerve conduction velocity in patients with diabetic neuropathy.

## Graphical abstract

Empagliflozin (10 mg/day for 20 weeks) improved glycaemic control, renal function, neuropathic symptoms and nerve conduction velocity in patients with type 2 diabetes and peripheral neuropathy, suggesting complementary metabolic, renal and neurophysiological benefits beyond glucose lowering.

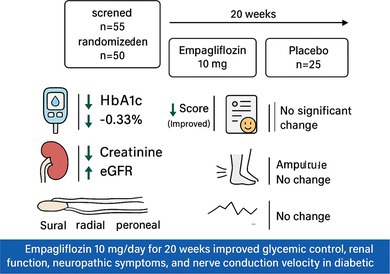

## 1 Introduction

Diabetes mellitus (DM) is the largest global epidemic of the 21st century and is associated with a range of complications, classified into microvascular and macrovascular categories [1, 2, 3, 4, 5]. Microvascular complications involve dysfunctions of the nervous system (diabetic neuropathy), renal system (diabetic nephropathy) and ocular system (diabetic retinopathy), while macrovascular complications include cardiovascular disease, stroke and peripheral vascular disease. Neuropathy, the most prevalent and widespread microvascular complication in DM, leads to the loss of sensory function, affecting different levels of the peripheral nerve (peripheral neuropathy), often resulting in pain and numbness, typically in the hands and feet [3]. Approximately 50% of individuals with diabetes are expected to develop diabetic neuropathy [5]. It is well established that DM arises from insulin resistance, which is linked to an inflammatory state characterised by elevated cytokine levels, leading to oxidative stress and endothelial cell dysfunction [5].

Anti‐diabetic medications play a crucial role in managing diabetes and preventing complications. These medications can potentially improve brain cell metabolism, which is particularly important for patients with neurodegenerative disorders associated with DM. Recent advancements in anti‐diabetic drugs, such as Dipeptidyl Peptidase‐4 (DPP4) inhibitors, glucagon‐like peptide‐1 receptor agonists and Sodium‐Glucose Cotransporter‐2 (SGLT2) inhibitors, have shown promising effects. These drugs help lower glycated haemoglobin, do not increase the risk of hypoglycaemia, and may contribute to weight loss [6].

SGLT2 inhibitors, for instance, reduce plasma glucose by preventing glucose reabsorption from the kidneys, regardless of the cell's mass or function. This results in the excretion of glucose in the urine, leading to a negative energy balance and weight loss. Additionally, they reduce blood pressure by inhibiting sodium absorption in the proximal kidney tubules. SGLT2 inhibitors also exert an insulin‐independent hypoglycaemic effect, lowering blood glucose even when insulin secretion is reduced. This mechanism improves insulin resistance, reduces glucose toxicity and enhances pancreatic cell function.

Moreover, SGLT2 inhibitors can improve mitochondrial function and insulin signalling in the brain, potentially protecting against cognitive decline by preserving synaptic flexibility. Additionally, they can increase blood ketone bodies, which may help modulate the pathological processes in neurodegenerative diseases [7, 8, 9].

Empagliflozin is an oral medication for lowering blood glucose, approved for clinical use in 2014 [10, 11]. It is part of the SGLT2 inhibitor class [12, 13, 14], which was introduced for managing type 2 diabetes mellitus (T2DM).

Empagliflozin specifically targets SGLT2 transporters [12, 13] rather than SGLT1 [13], which is crucial because SGLT2 is primarily located in the proximal tubules of the kidneys [12, 14], where approximately 90% of glucose reabsorption occurs [12]. In contrast, SGLT1 is found in other areas such as the intestines, heart and skeletal muscle.

Treatment with SGLT2 inhibitors (SGLT2I) may help manage autonomic neuropathy in diabetic patients by influencing the regulation of the vagal tone, heart rate and sympathetic nervous system. While numerous studies have demonstrated the effectiveness of SGLT2I in controlling blood glucose levels, further research is needed to assess their impact on improving diabetic peripheral neuropathy.

Due to the increasing prevalence of DM in Yazd province [15, 16, 17], and although empagliflozin has shown substantial benefits in glycaemic control, cardiovascular protection and renal outcomes, evidence regarding its effects on diabetic peripheral neuropathy remains limited [1, 18]. Most existing studies have focused on autonomic neuropathy or symptomatic measures of neuropathic pain, with few assessing electrophysiological parameters or validated neuropathy screening tools. Given the potential neuroprotective mechanisms of empagliflozin—such as reducing glucotoxicity, improving mitochondrial function and exerting anti‐inflammatory effects—a beneficial impact on peripheral nerve function is plausible [19, 20], yet clinical data are scarce. This study therefore seeks to address this knowledge gap by evaluating both clinical and electrophysiological outcomes in a randomised controlled trial.

## 2 Methods

### 2.1 Study Design and Randomisation

This double‐blind, randomised, controlled clinical trial was conducted on patients with DM. For the study, empagliflozin and placebo are prepared and packaged identically, with serial numbers kept confidential. Patients are randomly (using a random number table) assigned to either the control group (receiving placebo) or the treatment group (receiving 10 mg of empagliflozin daily to their existing regimen) [21]. Both groups continue their regular diabetes medication regimen.

### 2.2 Inclusion and Exclusion Criteria

The inclusion criteria for the study were a minimum of 3 years of diagnosed type 2 diabetes, confirmed by an endocrinologist, with a stable medication regimen for at least the past year, an HbA1c level of < 8%, and an age range of 30–60 years. Participants also had to have diabetic peripheral neuropathy (DPN) confirmed through neuropathy screening tests, and their diabetes diagnosis was based on fasting blood glucose (FBS) level greater than 126 mg/dL or a 2‐h oral glucose tolerance test (OGTT) plasma glucose level of < 200 mg/dL.

Exclusion criteria included pregnancy or breastfeeding, smoking, insulin use, hormone or oestrogen medications, antagonist drugs, dietary supplements and diseases that could affect study variables, such as liver or kidney disease, retinopathy, nephropathy, or acute or chronic inflammation. Additional exclusion factors included a history of hereditary or acquired neuropathy (e.g., hypothyroidism), vascular diseases (e.g., stroke, varicose veins), active foot infections, trauma, limb deformities or amputations, previous neuropathy treatment, and a history of neurological or psychiatric disorders, such as epilepsy or mood disorders.

### 2.3 Sample Size Calculation

The sample size for each group was 25 participants. The required sample size was calculated based on the variables HbA1c < 8%, BMI 32 + 1.5 kg/m^2^, with a 95% confidence level and 80% power, considering similar intervention studies [18, 19, 20] and in consultation with a statistical expert.

### 2.4 Methods of Diagnosis, Screening and Monitoring

Diagnosis of diabetic peripheral neuropathy in patients should follow standard protocols, involving clinical examinations, symptom assessment and electrodiagnostic testing. One reliable tool for screening is the Michigan Neuropathy Screening Instrument (MNSI), which uses a combination of patient history, clinical exams, monofilament testing and vibration threshold testing. A score of 4 or higher on the questionnaire or 2 or higher on the examination suggests neuropathy.

The MNSI included two parts: a 15‐question self‐report questionnaire and a physical examination. The questionnaire assigns points based on the patient's answers, with certain questions contributing differently to the score.

The physical examination assessed visible signs in the lower limbs (e.g., deformities, calluses, or infections) and included tests such as the Achilles reflex and vibration sensation. Additionally, the monofilament test checks for sensory loss, with points awarded based on the patient's responses.

Electrodiagnostic tests are also performed to evaluate nerve conduction in sensory and motor nerves. These tests help assess the extent of nerve damage.

The study also calculated the estimated Glomerular Filtration Rate (eGFR) for each participant before and after treatment to assess kidney function. After 20 weeks, the MNSI test and electrodiagnostic evaluations were repeated to measure the effects of empagliflozin on diabetic neuropathy.

Blood glucose monitoring is done at home, with regular check‐ins for potential hypoglycaemia. Blood samples were taken before the treatment and after 20 weeks to measure key indicators like HbA1c, blood glucose and creatinine levels.

### 2.5 Assessment of Patient Compliance (Patients Monitoring)

Patient compliance was monitored by pill counts at each follow‐up visit, and patients were instructed to return any unused medication. Compliance was > 90% in both groups. No adjustments to background anti‐diabetic medications were allowed during the 20‐week intervention, except in cases where blood glucose dropped below 80 mg/dL, in which case patients were referred to their treating physician. No participant required permanent medication changes, and thus background therapy remained stable throughout the study.

### 2.6 Statistical Analysis

Data analysis was performed using SPSS software, version 23. The primary analysis followed the intention‐to‐treat (ITT) principle, including all randomised patients who received at least one dose of the study medication. For participants with missing post‐intervention data, the last observation carried forward (LOCF) method was applied. A supportive per‐protocol (PP) analysis was also conducted, including only those patients who completed the 20‐week intervention without major protocol deviations; the results of the PP analysis were consistent with the ITT analysis.

Continuous variables were analysed using Paired Sample *t*‐tests (for within‐group comparisons) and Independent Sample *t*‐tests (for between‐group comparisons). A *p*‐value of < 0.05 was considered statistically significant.

### 2.7 Ethical Consideration

After obtaining written consent from patients, this study was approved by the Ethics Committee of Shahid Sadoughi University (IR.SSU.MEDICINE.1402.232). This study was registered in the Iranian Registry of Clinical Trials (IRCT20230919059469N1).

## 3 Results

**FIGURE 1:**
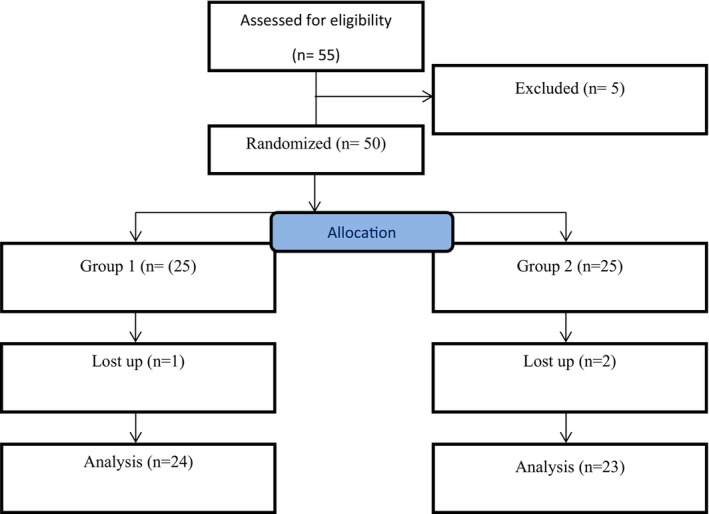
Consort flowchart.

The current study was conducted on 50 patients. The consort flowchart is shown in Figure 1.

The mean age of patients in intervention and control groups was 53.92 ± 3.02, and 54.70 ± 3.03 years, respectively (*p* = 0.383). The mean body mass index (BMI) in the two groups was 25.22 ± 1.62 and 25.38 ± 2.07 kg/m^2^, respectively (0.773). The results of laboratory tests before treatment in the two groups are shown in Table 1.

**TABLE 1:** The results of laboratory tests before treatment in the two groups.

Index	Control group	Intervention group	*p*
HbA1c	7.30 ± 0.35	7.42 ± 0.30	0.201
FBS	163.96 ± 10.94	167.00 ± 10.04	0.326
Cr	1.47 ± 0.39	1.37 ± 0.44	0.424
eGFR	53.47 ± 18.36	59.73 ± 20.40	0.276

As shown in Table 1, no significant difference was seen between the two groups before intervention (*p* > 0.05).

The results of the Michigan oral questionnaire and clinical examination before intervention between the two groups are shown in Table 2.

**TABLE 2:** Results of the Michigan oral questionnaire and clinical examination before intervention.

Score	Control group	Intervention group	*p*
Michigan Oral Questionnaire score	5.61 ± 1.07	5.79 ± 1.47	0.631
Michigan examination test score	3.37 ± 0.66	3.64 ± 0.74	0.186

As shown in Table 2, no significant difference was seen between the two groups before intervention (*p* > 0.05).

The results of initial electrodiagnostic studies in the two groups before intervention are shown in Table 3.

**TABLE 3:** The results of initial electrodiagnostic studies in the two groups before intervention.

Index	Control group	Intervention group	*p*
Sural nerve amplitude (μv)	8.77 ± 1.54	7.89 ± 2.62	0.167
Sural nerve delay (ms)	2.98 ± 0.14	3.00 ± 0.16	0.620
Sural nerve conduction velocity (m/s)	47.04 ± 2.09	46.75 ± 2.55	0.670
Radial nerve amplitude (μv)	20.00 ± 1.36	19.32 ± 1.98	0.179
Radial nerve delay (ms)	1.84 ± 0.11	1.85 ± 0.16	0.835
Radial nerve conduction velocity (m/s)	54.26 ± 3.10	54.29 ± 4.54	0.979
Common peroneal nerve amplitude (μv)	2.73 ± 0.63	2.51 ± 0.79	0.296
Common peroneal nerve delay (ms)	4.03 ± 0.45	4.20 ± 0.42	0.196
Common peroneal nerve conduction velocity (m/s)	42.26 ± 1.98	41.37 ± 1.63	0.101

As shown in Table 3, no significant difference was seen between the two groups before intervention (*p* > 0.05).

The results of laboratory tests before and after treatment in two groups are shown in Table 4.

**TABLE 4:** The results of laboratory tests before and after treatment in two groups.

Index	Control group (BT)	Control group (AT)	Intervention (BT)	Intervention (AT)	*p*
Hb A1c	7.30 ± 0.35	7.24 ± 0.29	7.42 ± 0.30	7.09 ± 0.32	0.001
FBS	163.96 ± 10.94	160.95 ± 8.82	167.00 ± 10.04	156.54 ± 11.37	0.001
Cr	1.47 ± 0.39	1.44 ± 0.39	1.37 ± 0.44	1.24 ± 0.39	0.001
eGFR	53.47 ± 18.36	53.85 ± 16.64	59.73 ± 20.40	65.76 ± 21.99	0.001

Abbreviations: AT, after treatment; BT, before treatment.

As seen in Table 4, the results of fasting blood glucose and haemoglobin A1c in the group receiving empagliflozin showed a significant improvement compared to baseline, reflecting a reduction in both blood glucose and haemoglobin A1c levels. Blood creatinine levels also showed a significant decrease, with a P value of 0.001. Additionally, eGFR was calculated before and after treatment for both groups, and the changes in eGFR compared to baseline were assessed. The reduction in eGFR was significantly greater in the treatment group compared to the control group.

The results of initial electrodiagnostic studies between the two groups (before and after intervention) are shown in Table 5.

**TABLE 5:** The results of initial electrodiagnostic studies in the two groups (before and after intervention).

Index	Control group (BT)	Treatment group (AT)	Control group (BT)	Treatment group (AT)	*p*
Sural nerve amplitude (μv)	7.89 ± 2.62	7.84 ± 2.65	8.77 ± 1.54	8.79 ± 1.45	0.628
Sural nerve delay (ms)	3.00 ± 0.16	2.85 ± 0.16	2.98 ± 0.14	3.02 ± 0.15	0.004
Sural nerve conduction velocity (m/s)	46.75 ± 2.55	49.25 ± 2.73	47.04 ± 2.09	46.34 ± 2.08	0.004
Radial nerve amplitude (μv)	19.32 ± 1.98	19.27 ± 2.01	20.00 ± 1.36	19.93 ± 1.41	0.120
Radial nerve delay (ms)	1.85 ± 0.16	1.79 ± 0.15	1.84 ± 0.11	1.85 ± 0.12	0.005
Radial nerve conduction velocity (m/s)	54.29 ± 4.54	56.12 ± 4.58	54.26 ± 3.10	54.17 ± 3.55	0.002
Common peroneal nerve amplitude (μv)	2.51 ± 0.79	2.50 ± 0.81	2.73 ± 0.63	2.72 ± 0.66	0.640
Common peroneal nerve delay (ms)	4.20 ± 0.42	3.97 ± 0.46	4.03 ± 0.45	3.99 ± 0.48	0.000
Common peroneal nerve conduction velocity (m/s)	41.37 ± 1.63	43.45 ± 2.53	42.26 ± 1.98	42.52 ± 2.40	0.000

Abbreviations: AT, after treatment; BT, before treatment.

After 20 weeks of intervention and data collection, a significant increase in the nerve conduction velocity of the sural, radial and common peroneal nerves was observed. Additionally, the latency in all three nerves decreased, but the amplitude did not show a significant difference compared to before the intervention.

The results of the Michigan Oral questionnaire and clinical examination between the two groups before and after intervention are shown in Table 6.

**TABLE 6:** The results of the Michigan oral questionnaire and clinical examination between the two groups before and after intervention.

Index	Intervention group (BT)	Intervention group (AT)	Control group (BT)	Control group (AT)	*p*
Michigan Oral Questionnaire score	5.79 ± 1.47	4.92 ± 1.06	5.61 ± 1.07	5.43 ± 1.12	0.001
Michigan examination test score	3.64 ± 0.74	3.52 ± 0.71	3.37 ± 0.66	3.32 ± 0.68	0.073

Abbreviations: AT, after treatment; BT, before treatment.

As observed, the Michigan oral exam score in the group that received empagliflozin showed a significant difference compared to their baseline, indicating a decrease in the oral exam score and an improvement in symptoms as reported by the patient. The Michigan clinical examination score, both before and after treatment, was also compared for each individual in both groups. With a P value of 0.073, the results did not show a significant difference.

## 4 Discussion

In our study, we investigated the effect of adding empagliflozin to the medication regimen on peripheral neuropathy in patients with type II diabetes. The results showed significant reductions in fasting blood sugar and haemoglobin A1c after 20 weeks of empagliflozin use. Empagliflozin is known as a glucose‐lowering medication. Notably, we did not alter other medications that participants were taking, and we only referred patients to their treating physician if their blood sugar dropped below 80 mg/dL. The average decrease in fasting blood sugar was 10.46 mg/dL, and the reduction in haemoglobin A1c was 0.33%.

Although the reductions in HbA1c (0.33%) and fasting blood glucose (~10 mg/dL) observed in our study may appear modest compared to larger trials, they are nonetheless clinically meaningful. Evidence from the prospective diabetes study in England, Scotland and Northern Ireland demonstrated that every 1% reduction in updated mean HbA1c was associated with a 21% reduction in the risk of any diabetes‐related endpoint (95% CI: 17%–24%; *p* < 0.0001), a 21% reduction in diabetes‐related mortality (95% CI: 15%–27%; *p* < 0.0001), a 14% reduction in myocardial infarction (95% CI: 8%–21%; *p* < 0.0001), and a 37% reduction in microvascular complications (95% CI: 33%–41%; *p* < 0.0001) [22].

In a study by Mazhar et al. the impact of empagliflozin and dapagliflozin on type II diabetes treatment was evaluated. After 12 weeks, the empagliflozin group experienced reductions of 51.6 mg/dL in fasting blood sugar and 2.18% in haemoglobin A1c, while the dapagliflozin group saw reductions of 61.4 mg/dL in fasting blood sugar and 1.4% in haemoglobin A1c [23]. Beyond differences in study duration and baseline HbA1c, several additional factors may explain these discrepancies. First, our trial excluded patients with HbA1c ≥ 8% or those requiring insulin, resulting in a study population with better baseline glycaemic control and less potential for large reductions. Second, patient adherence may vary across studies, and the double‐blind design of our trial may have minimised behavioural or placebo‐related improvements. Third, dosing regimens differed, as some prior trials used titration to 25 mg empagliflozin, whereas we employed a fixed 10 mg daily dose to balance efficacy and safety. Finally, neuropathy was assessed in our study using both the MNSI and electrophysiological testing, while other studies applied symptom‐based questionnaires (e.g., DN‐4, DNS) that may be more sensitive to short‐term symptomatic changes. Collectively, these methodological and clinical differences may account for the discrepancy in outcomes.

A 2022 study by Deepac et al. examined the effects of four sodium‐glucose cotransporter‐2 (SGLT2) inhibitors—empagliflozin, dapagliflozin, canagliflozin and remogliflozin—on type II diabetes treatment. After 24 weeks of treatment, the reductions in fasting blood sugar were 54.16, 54.79, 55.62 and 52.12 mg/dL, respectively, while haemoglobin A1c decreased by 2.87%, 2.74%, 3.08% and 2.79%, respectively [24].

In a 2018 retrospective study, the addition of empagliflozin, dapagliflozin and canagliflozin to the oral medication regimen of patients (who were not using insulin) over the past 6 months was examined [25]. The results showed a significant reduction in fasting blood sugar by 63.65 mg/dL and a significant decrease in haemoglobin A1c by 1.63% in all three groups. In comparison, these reductions in fasting blood sugar and haemoglobin A1c were greater than in our study. One possible explanation for this difference is the shorter duration of our study compared to the other two. Another reason could be the self‐adjustment of other medications by patients during the treatment process, as this confounding factor was not controlled or eliminated in our study. A third reason may be the higher baseline levels of fasting blood sugar and haemoglobin A1c in the other studies. In our study, patients who required insulin or had haemoglobin A1c levels above 8% were excluded. In the three studies, the initial fasting blood sugar levels were 177 ± 44.6, 193.93 ± 22.52 and 198.21 ± 38.21 mg/dL, and the initial haemoglobin A1c levels were 9.8% ± 2.2%, 11.6% ± 1.76% and 8.92% ± 1.47%, respectively, all of which were higher than the baseline levels in our study (165.51 ± 10.49 mg/dL, 7.36% ± 0.33%).

In our study, to assess the effect of empagliflozin on kidney function, blood creatinine levels were measured before and after 20 weeks of treatment, showing a significant decrease. Additionally, eGFR (estimated Glomerular Filtration Rate) was calculated for all patients before and after the intervention, and the reduction in eGFR was compared between the two groups, showing a significant difference. The treatment group had a greater reduction in eGFR, indicating improvement in diabetic nephropathy.

In the 2023 study by Mikkle Jürgens et al. the effect of empagliflozin on glomerular filtration and diabetic nephropathy was examined. The initial measured GFR (mGFR) was 72.3 ± 23.5 in the empagliflozin group and 73.9 ± 22.8 in the control group. After 13 weeks of empagliflozin treatment, the reduction in eGFR was −8.8 in the empagliflozin group and −0.9 in the control group, showing a significant difference. In our study, the reduction in eGFR was −6.02 ± 4.21 in the treatment group and −0.37 ± 4.54 in the control group, demonstrating a greater reduction in the treatment group and improvement in nephropathy [26].

In our study, diabetic peripheral neuropathy was assessed using the Michigan Neuropathy Screening Instrument (MNSI) and electrophysiological testing. The MNSI consists of two parts: a self‐administered questionnaire and a clinical examination. The self‐administered Michigan test in the treatment group showed a significant reduction from 5.79 ± 1.47 to 4.92 ± 1.06, while no significant difference was observed in the control group. However, the clinical examination did not reveal a significant difference between the two groups.

In a 2022 study, the effect of dapagliflozin on cardiac autonomic neuropathy was evaluated. The results showed improvements in heart rate variability and a reduction in the frequency of ventricular arrhythmias. Despite maintaining stable blood sugar and haemoglobin A1c levels in both the treatment and control groups, the observed changes suggest a direct impact of the drug on cardiac autonomic function [27].

A 2022 cohort study by Yang et al. found that the incidence of amputations was lower in the group using SGLT2 inhibitors compared to the group using DPP‐4 inhibitors [28]. However, a 2019 meta‐analysis indicated that the rate of amputations in patients using SGLT2 inhibitors did not show a significant reduction compared to the general population.

In a 2021 study, the effectiveness of empagliflozin and vitamin D supplementation on diabetic neuropathy was evaluated. This study used the DN‐4 score, which involves fewer questions and assessments than the MNSI. Although symptom improvement and a reduction in neuropathy scores were observed in the empagliflozin‐only group, the difference was not statistically significant compared to the control group. However, the combined use of empagliflozin and vitamin D supplementation showed a significant improvement [1].

In the study by El‐Haggar et al., the effect of empagliflozin on type 2 diabetic neuropathy was assessed after 3 months of treatment. Several indices were used to evaluate neuropathy in this study, including the Diabetic Neuropathy Symptom (DNS) score, electrophysiological testing (which involved sensory sural nerve and motor common peroneal and posterior tibial nerves on both sides), and the Brief Pain Inventory Short Form (BPI‐SF) score to assess patient pain levels [18].

In this study, significant improvements were observed in most factors, such as Amplitude, Latency and NCV (nerve conduction velocity) for the sural sensory nerve, common peroneal motor nerve and posterior tibial motor nerve. In contrast, our study found significant increases in nerve conduction velocity for all three nerves (sural, radial and common peroneal), along with a significant reduction in latency. However, no significant difference was observed in amplitude.

In this study, unlike ours, the DNS score, which reflects the clinical symptoms of the patients, did not show a significant improvement. Additionally, their study did not observe a significant reduction in haemoglobin A1c, leading to the conclusion that the independent effect of empagliflozin contributed to the improvement in electrophysiological changes.

## 5 Conclusion

Based on the findings of this double‐blind clinical trial, empagliflozin can effectively reduce haemoglobin A1c and fasting blood glucose levels. It also decreases plasma creatinine levels and increases eGFR. Additionally, empagliflozin led to an improvement in the clinical symptoms of the patients; however, no significant differences were observed in their clinical examination scores. In electrophysiological assessments, empagliflozin improved nerve conduction velocity, but no changes were seen in other parameters.

### 5.1 Limitations of the Study

This study was conducted over 20 weeks; a longer duration might have yielded more comprehensive results. Another limitation of the study design was the absence of blood glucose monitoring during this period. In addition, the relatively small sample size—although calculated to provide adequate power for the primary outcomes—may reduce the generalizability of the findings. The single‐centre design further limits external validity. Therefore, larger multicentre randomised controlled trials with longer follow‐up are warranted to validate and extend these results.

## Author Contributions

M.V., A.G.‐A., R.A. and A.K. conceived of the presented idea and developed the theory and wrote the primary draft and supervised the project. F.M.M. and M.Z.B. wore the result and discussion sections and contributed to the perform manuscript and approved the final version and edited the manuscript.

## Conflicts of Interest

The authors declare no conflicts of interest.

## Data Availability

The data that support the findings of this study are available on request from the corresponding author. The data are not publicly available due to privacy or ethical restrictions.
